# Determination of Stark parameters by cross-calibration in a multi-element laser-induced plasma

**DOI:** 10.1038/srep25609

**Published:** 2016-05-12

**Authors:** Hao Liu, Benjamin S. Truscott, Michael N. R. Ashfold

**Affiliations:** 1 School of Chemistry, University of Bristol, Bristol, BS8 1TS, UK

## Abstract

We illustrate a Stark broadening analysis of the electron density *N*_e_ and temperature *T*_e_ in a laser-induced plasma (LIP), using a model free of assumptions regarding local thermodynamic equilibrium (LTE). The method relies on Stark parameters determined also without assuming LTE, which are often unknown and unavailable in the literature. Here, we demonstrate that the necessary values can be obtained *in situ* by cross-calibration between the spectral lines of different charge states, and even different elements, given determinations of *N*_e_ and *T*_e_ based on appropriate parameters for at least one observed transition. This approach enables essentially free choice between species on which to base the analysis, extending the range over which these properties can be measured and giving improved access to low-density plasmas out of LTE. Because of the availability of suitable tabulated values for several charge states of both Si and C, the example of a SiC LIP is taken to illustrate the consistency and accuracy of the procedure. The cross-calibrated Stark parameters are at least as reliable as values obtained by other means, offering a straightforward route to extending the literature in this area.

Quantitative determinations of the electron density *N*_e_ and temperature *T*_e_—or, more generally, the electron energy distribution function (EEDF)—are fundamental to experimental plasma physics. The absolute values of these plasma parameters are customarily measured using Langmuir probe, Thomson scattering, or laser interferometry diagnostics. As an indirect, invasive technique capable of only limited spatial and temporal resolution, the former is often difficult to apply in practice, while the laser-based methods are preferred as benchmarks but require complex instrumentation and have unintended probe-induced heating effects as a recognized concern^1^,^2^.

Optical emission spectroscopy (OES) offers another, highly practical and truly non-invasive route to obtaining spatially and temporally resolved values of *N*_e_ and *T*_e_, albeit one for which the apparent simplicity of the experiment sometimes belies the difficulty of making a valid measurement. Customary analyses usually rely on the assumption of local thermodynamic equilibrium (LTE)—that is, a Maxwellian EEDF and Boltzmann population ratios—to evaluate *T*_e_ from the relative intensities of (atom or ion) emission lines according to the “Boltzmann plot” method and, given this information, *N*_e_ from the Stark-broadened widths^3^,^4^,^5^. Other diagnostics that typically assume LTE include the use of the line-to-continuum intensity ratio or Planck’s law for *T*_e_, which can then be used to recover *N*_e_ via absolute spectral radiance measurements^6^. Alternatively, *N*_e_ and *T*_e_ can be obtained simultaneously, given only the line intensities, from Saha–Boltzmann population analysis^7^, or only from the Stark widths if several suitably chosen lines are considered together^8^,^9^. However, although spectroscopic determinations of the electron parameters inherently require a (locally) homogenous, quasi-static plasma, such exploitation of only a minimal subset of the available information leaves no standard according to which the applicability of these assumptions may be tested, and hence can potentially lead to ambiguous or inaccurate determinations^10^. A more subtle, although considerably more robust, approach is to seek the best fit to experiment of a self-consistent model spectrum, to which *N*_e_ and *T*_e_ are cast as parameters. The calculation of Stark-broadened line profiles requires that the relevant transitions be correctly assigned (in order for the upper and lower state energies and degeneracies to be known), and that the resulting observed lines have tabulated values for the Stark parameters (width *w* and shift *d*) over the density and temperature range of interest. Nonetheless, for many elements, there are only limited means available for the production of plasmas with widely varying *N*_e_ and *T*_e_ and containing ions in several different charge states, which complicates calibration and so limits the availability of these values in the literature.

Laser-induced plasmas (LIPs) are, in principle, an attractive source for the measurement of Stark parameters owing to their straightforward generation from any material. It is, however, difficult to produce LIPs with well-defined plasma parameters, and further challenges are posed by their spatial and temporal inhomogeneity. Although these problems are mitigated and a uniform, high density (an essential precondition for LTE) can be expected for LIPs produced in an ambient medium, the attainable range of densities and temperatures, as well as the ion populations, are limited compared with the same discharges in a vacuum environment (which, however, may be far from LTE). LIPs are thus generally inadequate as primary Stark broadening standards, but represent excellent secondary standards for determination of *w* and *d*, especially for the more highly charged ions, by calibration transfer if *N*_e_ and *T*_e_ can be measured reliably and free of assumptions regarding LTE. Indeed, while these quantities can certainly be obtained from analysis of non-LTE spectral line shapes given Stark widths and shifts determined under the appropriate plasma conditions^11^,^12^, there is a paucity of data for the latter measured using benchmark methods. Most of the Stark parameters discussed in key theoretical works^13^ and listed in critical compilations of experimental data^14^,^15^,^16^ have been quoted for values of *N*_e_ and *T*_e_ established under assumptions applicable only to LTE conditions.

Experimentally, strong gradients of density and temperature in LIPs mandate spatially and temporally resolved measurements, and shot-to-shot intensity variations lead to a strong preference for multi-channel detectors. Our recent time-gated, imaging spectroscopy study of the 1064 nm, nanosecond pulsed laser ablation (PLA) of Si demonstrated successful non-LTE measurements of *N*_e_ and *T*_e_ for a LIP rapidly expanding into vacuum^11^, and showed that the electron characteristics obtained by analysing spectral lines of one emitter can in fact be used to establish Stark parameters for another, collocated species. In that work, we were motivated by the dearth of published values of *w* and *d* for Si IV transitions. Here, using a silicon carbide LIP as an example, we confirm that the same procedure can accurately reproduce known results for one element from those tabulated for another, illustrating the consistency of the approach and suggesting the opportunity for a straightforward, significant expansion of the literature. A comprehensive database of non-LTE Stark broadening parameters would greatly improve the applicability of such analyses, not only to LIPs in vacuum but also to other low-density plasmas, such as those of astrophysical interest.

## Results and Discussion

We employ SiC as an example of a material for which the LIP contains two species (C^+^ and Si^+^, henceforth C II and Si II) with emission lines at similar wavelengths, for both of which there exist well-determined Stark broadening parameters independent of any assumptions regarding LTE^15^. A 532 nm, nanosecond pulsed laser was focused onto the surface of a SiC target mounted inside a high vacuum chamber. Spatially resolved LIP plume emission spectra were recorded for different time delays following PLA, yielding time-resolved images with one spatial and one spectral axis.

**Figure 1:**
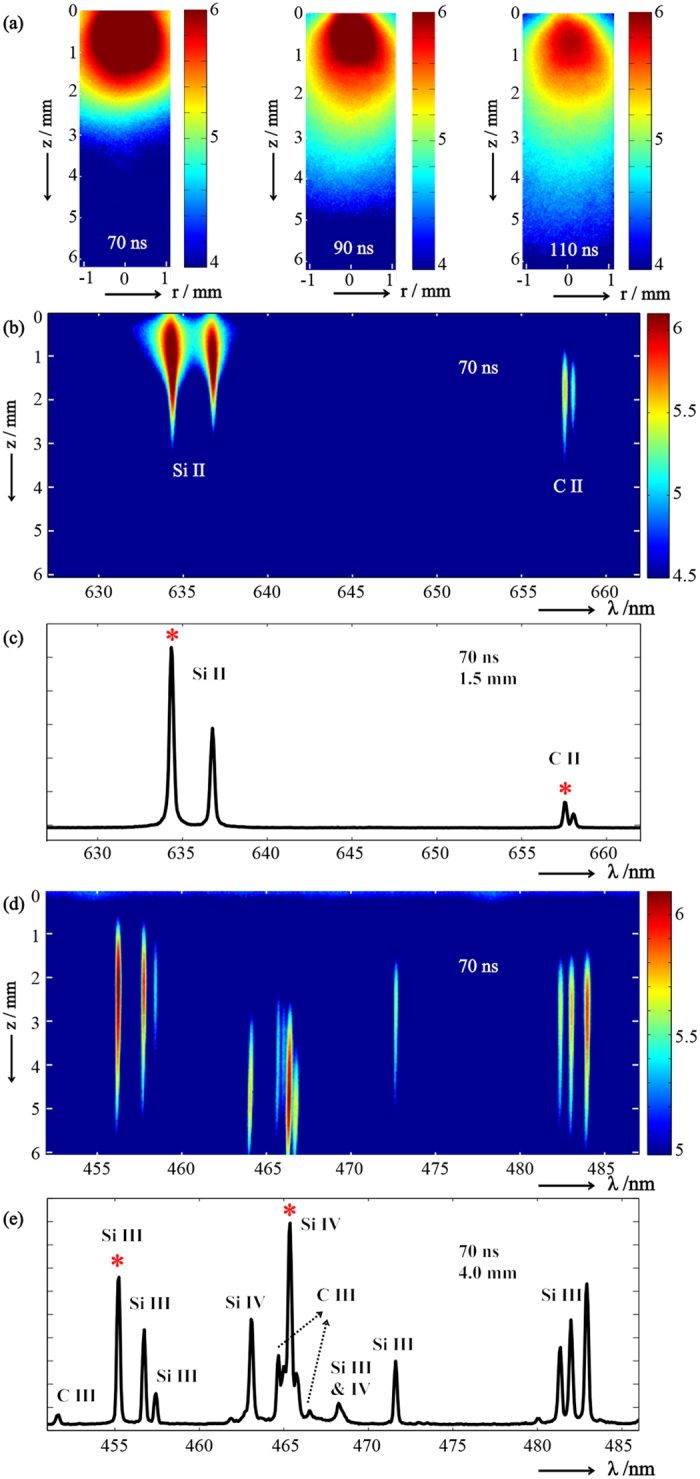
Optical emission measurements of a SiC LIP expanding into vacuum: (**a**) spatial distribution of the total visible (400 ≤ *λ* ≤ 800 nm) emission for delays Δ*t* = 70, 90, and 110 ns; (**b**) *I*(*z*, *λ*; Δ*t* = 70 ns) image and (**c**) corresponding *I*(*λ*; *z* = 1.5 mm) spectrum illustrating Si II and C II doublets. Additional Si transitions are highlighted in the *I*(*z*, *λ*; Δ*t* = 70 ns) image (**d**) and *I*(*λ*; *z* = 4.0 mm) spectrum (**e**). Lines analysed in the present work are marked with an asterisk here and in Table 1. The base-10 logarithmic false-colour intensity scale is shown to the right of each image.

For orientation, Fig. 1(a) shows spatially resolved images of the total emission in the wavelength range 400 ≤ *λ* ≤ 800 nm measured for time delays Δ*t* = 70, 90 and 110 ns following PLA of the SiC target with incident irradiance *ϕ* ≈ 20 GW cm^−2^. These and all subsequent images employ a logarithmic false-colour intensity scale, shown at the right of each panel. Such broadband visible images illustrate the propagation of the LIP away from the target (defined as distance *z* = 0) and emphasize its symmetry about the surface normal at the point of ablation (*r* = 0). Information on the species responsible for this emission is contained within the spatially *and* spectrally resolved images *I*(*z*, *λ*; Δ*t* = 70 ns), Fig. 1(b,d), where now the single spatial axis is chosen as nominally (*z*, *r* = 0) and the respective wavelength ranges are 450 < *λ* < 490 nm and 625 < *λ* < 665 nm. Here, the extended *z*-profile of each emission feature reflects the range of (average) velocities with which the associated carrier has propagated prior to the measurement. Horizontal cuts through such images are the wavelength-resolved spectra *I*(*λ*; *z*, Δ*t*) of the species contributing to the emission at given *z*, which demonstrate more clearly the line shapes. Example spectra are shown in Fig. 1(c) for *z* = 1.5 mm and (e) for *z* = 4.0 mm, where in both cases the line assignments were obtained from the NIST Atomic Spectra Database^17^.

**Figure 2:**
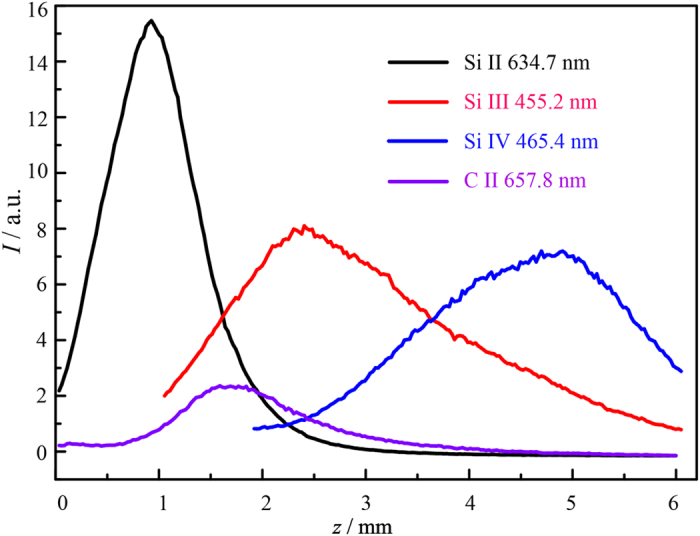
Spatial profiles *I*(*z*, Δ*t* = 70 ns) for the Si II, Si III, Si IV and C II transitions marked with an asterisk in Fig. 1 and Table 1, showing the regions of overlap between the various species.

As in our recent study of the 1064 nm PLA of Si^11^, we draw particular attention to the spatial fractionation of the various species within a LIP expanding into vacuum. The *z*-distributions of the emitters for any given Δ*t* are available from vertical cuts through the spatiospectral images, of which the family *I*(*z*; *λ*, Δ*t* = 70 ns) is shown in Fig. 2, with *λ* as given in Table 1 for Si II, Si III, Si IV and C II. That all lines associated with a given species and charge state show the same spatial profile affords a straightforward, visual route to identifying the carrier of each emission, even if assignments are ambiguous or unavailable. Furthermore, cross-calibration may be performed for any two or more species among which there exists a region of overlap. This overlap persists over a considerable span of *z* and *t*, thereby providing the opportunity to sample widely varying values of *N*_e_ and *T*_e_, and also offering a strong validation of internal consistency, i.e. that all of *N*_e_(*z*; Δ*t*), *T*_e_(*z*; Δ*t*), *w*, and *d* are recovered correctly for each species. Such a rigorous test of transferability is important since these determinations are subject to certain restrictions that cannot be assumed to be satisfied *a priori*. Firstly, the species must be actually collocated within a uniform environment, not only in *z* but also in *r*, which is given only if the depth of field of the imaging optics is small relative to the lateral extent over which inhomogeneities, in either the species distributions or the plasma parameters, become significant. And secondly, the plasma must not be so dense that radiation at the wavelengths of interest is unable to escape from the imaged volume, although it need not be optically thin if, as here, self-absorption is taken into account. While these conditions will be seen to be fulfilled in the present case from the mutually identical *N*_e_ and *T*_e_ values recovered from each species, we emphasize that reliable determinations of unknown Stark parameters require careful attention to these limitations, which may necessitate different experimental conditions or a more sophisticated apparatus than those used in this work.

Here, as previously^11^, *N*_e_ and *T*_e_ are determined by fitting using the line shape


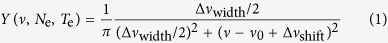


where *ν*_0_ is the field-free centre frequency of the transition (with corresponding wavelength *λ*_0_) and Δ*ν*_width_ and Δ*ν*_shift_ are the FWHM width and shift of the Stark-broadened line. These are further given by


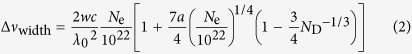


and


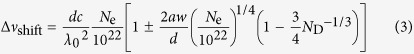


where *w* and *d* are the electron impact parameters (with units of length) for the Stark width and shift, *a* is a dimensionless ion impact parameter (which, for any given species, is nearly constant^18^,^19^), *N*_e_ is the electron density (in m^−3^), and *N*_D_ is the number of particles in the Debye sphere.

There has been much prior discussion of the applicability of LTE to LIPs over a range of densities^1^,^18^,^20^,^21^, but as established in our earlier study^11^, LIPs expanding into high vacuum are largely far from LTE. Consequently, the Saha–Boltzmann ionization balance does not apply, and therefore, as mentioned above, a key feature of the present analysis is that the model spectrum is calculated with populations given by a non-LTE generalization of the Saha equation based on a Druyvesteyn (rather than Maxwellian) EEDF. Such an EEDF has often been employed for the analysis of plasmas out of LTE, especially in the case of so-called coronal (collisional–radiative) equilibrium^22^,^23^,^24^. Indeed, a family of EEDFs has been proposed^22^ having the general form


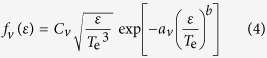


where *C*_*v*_ and *a*_*v*_ are dimensionless coefficients and *b* ≥ 1 is a real-valued parameter permitting reproduction of the Maxwell–Boltzmann (*b* = 1) and Druyvesteyn (*b* = 2) distributions as special cases. Here, to ensure internal consistency, we employ tabulated energy levels^17^ to explicitly compute all population ratios beginning with the ground state neutrals, and equate the electron density with the total ionic charge. Our comparison among the cases *b* = 1, 2, and 3 for a Si LIP expanding *in vacuo*^11^ found that a Druyvesteyn EEDF provided a fit to experiment that was consistently either equivalent to, or better than, either of the alternatives.

**Figure 3:**
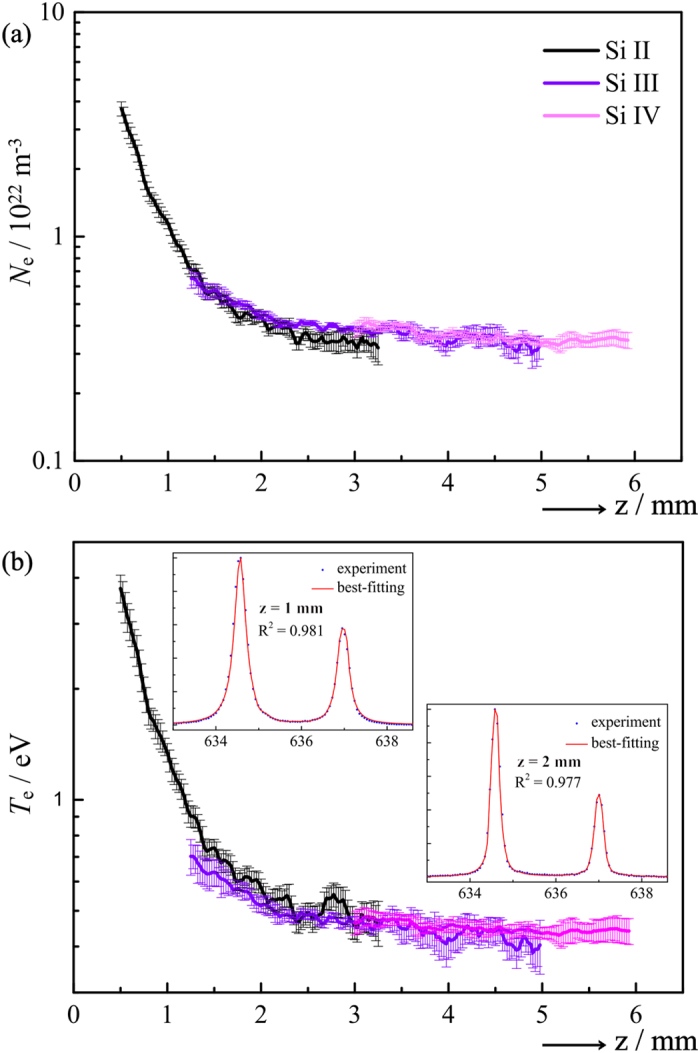
(**a**) *N*_e_(*z*) and (**b**) *T*_e_(*z*) measured for Δ*t* = 70 ns from the Stark-broadened Si II, Si III and Si IV lines marked with an asterisk in Fig. 1 and Table 1. The insets in (**b**) illustrate the fit of the Si II model spectrum to experiment at *z* = 1.0 and 2.0 mm.

We start by mapping *N*_e_(*z*; Δ*t* = 70 ns) and *T*_e_(*z*; Δ*t* = 70 ns) using Stark parameters, available in the existing literature, for the transitions of Si II at 634.710 and 637.136 nm^15^,^25^, Si III at 455.262, 456.784, and 457.476 nm^26^, and Si IV at 465.432 nm^11^. These values, listed in Table 1, have all been determined (by us in the latter case, using cross-calibration) in relation to *N*_e_ measurements free of any assumption regarding LTE. As Fig. 3 shows, the different expansion velocities of the silicon cations cause each to report on a particular range of *z*, but the values of the plasma parameters returned by the various charge states in regions of spatial overlap are reassuringly similar. Representative fits of the calculated Si II spectrum to experiment, illustrated in the insets to Fig. 3 (b), yield coefficients of determination *R*^2^ = 0.981 at *z* = 1 mm and 0.977 at *z* = 2 mm. Analyses of all three line shapes taken together are able to track an order-of-magnitude fall in both *N*_e_ and *T*_e_ over the range 0.5 ≤ *z* ≤ 6.0 mm.

**Figure 4:**
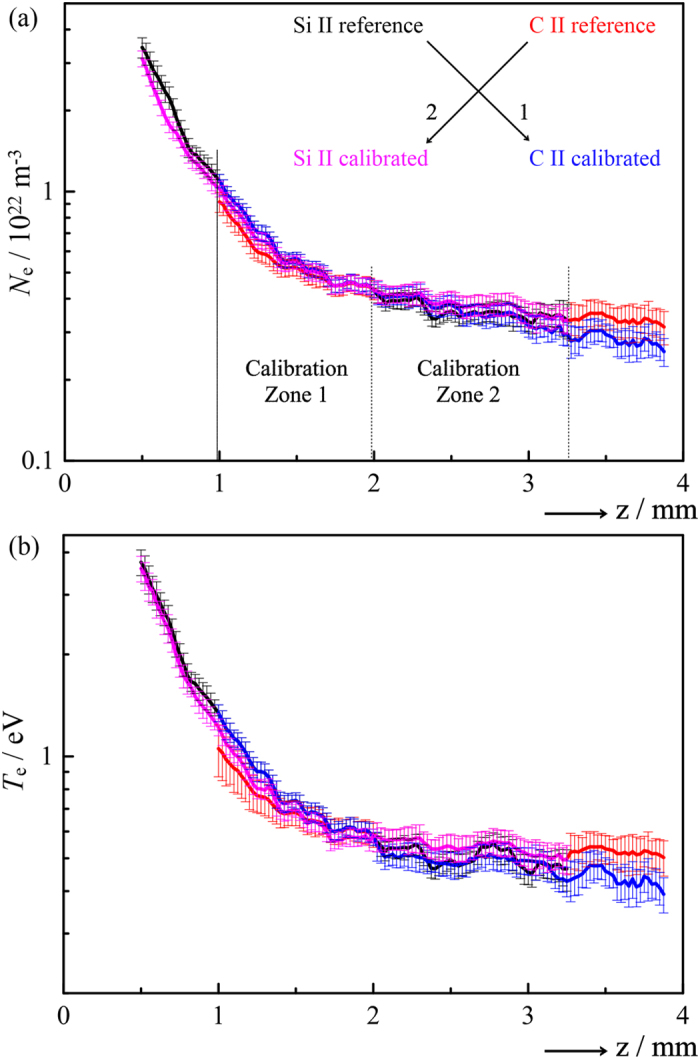
(**a**) *N*_e_(*z*) and (**b**) *T*_e_(*z*) for Δ*t* = 70 ns showing the effect of cross-calibration between the Si II and C II transitions marked with an asterisk in Fig. 1 and Table 1. In calibration zone 1 (1.0 ≤ *z* ≤ 2.0 mm), the Stark parameters for C II were derived based on *N*_e_ and *T*_e_ values determined using literature Stark parameters for Si II (black line). The cross-calibrated parameters were then used to obtain *N*_e_(*z*) and *T*_e_(*z*) from C II (blue line). The opposite procedure was applied in calibration zone 2 (2.0 ≤ *z* ≤ 3.2 mm), using the known Stark parameters for C II (red line) to obtain those for Si II, according to which *N*_e_(*z*) and *T*_e_(*z*) were replotted (pink line).

The spectral region covered in Fig. 1(b) was selected, in part, because of the availability of appropriate Stark parameters^13^,^25^,^27^ for both of the nearby Si II (634.710 nm) and C II (657.805 nm) transitions, which are marked with an asterisk in Fig. 1(c) and Table 1. Emissions from both species coincide for 1.0 ≤ *z* ≤ 3.2 mm in the Δ*t* = 70 ns image. This establishes our cross-calibration range, which is further divided into two zones. Within zone 1, 1.0 ≤ *z* ≤ 2.0 mm, we obtained *N*_e_(*z*) and *T*_e_(*z*) from Si II. Transfer of these values to C II yielded best-fit estimates of *w*^C II^ and *d*^C II^. The inverse procedure was applied in zone 2, 2.0 ≤ *z* ≤ 3.2 mm; that is, plasma parameters obtained from C II were used to derive *w*^Si II^ and *d*^Si II^. Each calibration zone further serves as the validation range for the other, i.e. it provides for an unbiased comparison between the values of *N*_e_ and *T*_e_ derived with either species as the primary standard. Evidently, both sets of cross-calibrated Stark parameters (Table 1) accurately reproduce the literature values, and *N*_e_(*z*) and *T*_e_(*z*) (Fig. 4) are highly consistent, not only in the opposite zone but even when extrapolated into the peripheral ranges *z* < 1.0 mm and *z* > 3.2 mm, where Si II and C II do not coexist. Indeed, the determinations coincide everywhere to within statistical error, even despite the limited accuracy with which the C II line widths could be determined due to the comparatively low-resolution spectrograph employed here. To validate the reproducibility of the cross-calibrated values, the experiment was repeated in its entirety under closely comparable conditions, but without attempting to replicate exactly the first set of measurements. The results, marked with a double dagger in Table 1, were statistically identical to those obtained originally, and the agreement between *N*_e_ and *T*_e_ as sampled by Si II and C II (not shown) was fully equivalent to that displayed in Fig. 4.

**Figure 5:**
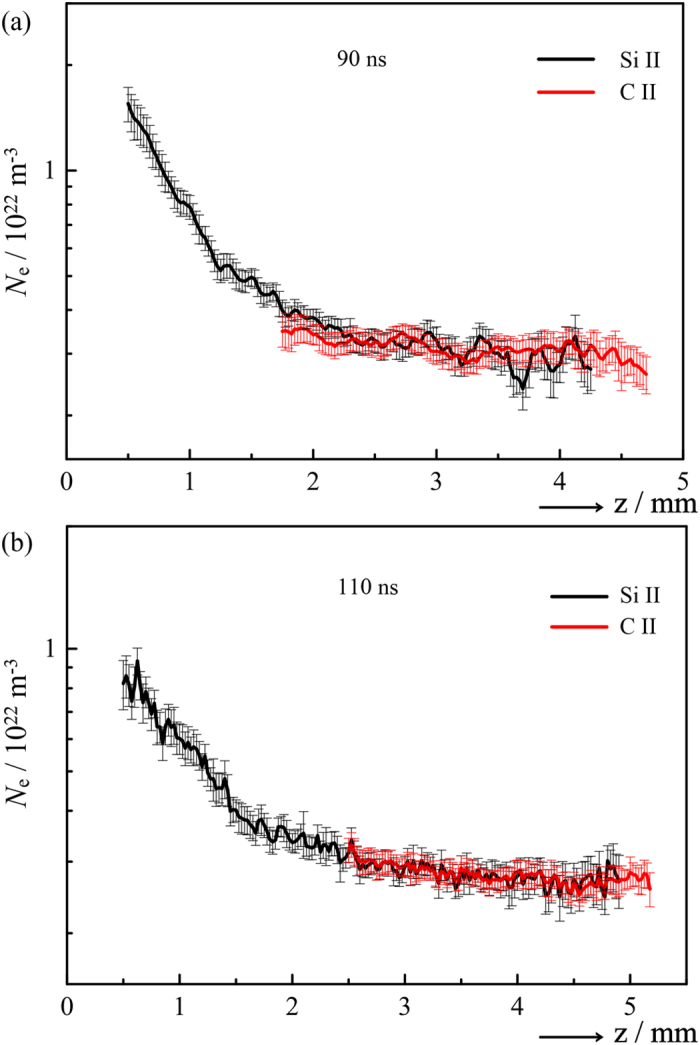
*N*_e_(*z*) derived from the Si II and C II line shapes, (**a**) for Δ*t* = 90 ns, using literature Stark parameters for Si II and cross-calibrated values for C II, and (**b**) for Δ*t* = 110 ns, using literature C II and cross-calibrated Si II Stark parameters. In both cases, the cross-calibrated parameters are those based on the Δ*t* = 70 ns data as in Fig. 4 (listed in Table 1), and not re-calibrated for the longer delays.

It should be noted that the properties of the LIP are not in any sense guaranteed, and so such a procedure not only grants access to previously unknown Stark parameters but also, through weak assumptions and strong self-consistency requirements, can provide improved confidence in Stark broadening as a diagnostic under the present experimental conditions. The finding that the local plasma parameters do not depend (inadvertently or otherwise) on the cation by which they are sampled warrants further testing in particular. To this end, Fig. 5 shows *N*_e_(*z*) distributions obtained by applying (a) the literature Si II and cross-calibrated C II Stark parameters to emission data recorded for Δ*t* = 90 ns, and (b) the literature C II and cross-calibrated Si II parameters to the corresponding data measured for Δ*t* = 110 ns. In both cases, the parameters derived for Δ*t* = 70 ns lead to commensurate absolute values of *N*_e_(*z*) in the overlap region, indicating that the cross-calibrated values are transferable and that the local environments of Si II and C II remain comparable as the LIP expands. We stress that such a conclusion may not hold in other scenarios (e.g. for LIPs containing H atoms^8^) given the simple one-temperature model, linear temperature dependence, and constant ion impact contribution to broadening assumed here. However, the constraints placed on the calculation of the model spectrum are such as to render any departure from the given assumptions easily detectable, and appropriate generalizations may be incorporated as necessary.

In summary, a SiC LIP produced by 532 nm, nanosecond PLA has been investigated in vacuum by time-gated, space- and wavelength-resolved imaging of the accompanying visible emission. Analysis of Stark-broadened C II, Si II, Si III and Si IV line shapes, crucially without incorporating any assumptions regarding LTE, has provided determinations of the time-evolving absolute values of *N*_e_(*z*) and *T*_e_(*z*) near the central axis of the plasma plume that are fully mutually consistent among the species. We have thus shown that, given well-characterised Stark parameters for at least one observed line, it is possible to derive hitherto unknown parameters for other collocated emitters even in the highly inhomogeneous non-LTE environment of a LIP. The ease of generation, wide variation of *N*_e_ and *T*_e_, and the possibility of multiple independent evaluations of each quantity for this plasma source all suggest a straightforward route to expanding the literature of Stark broadening parameters determined without recourse to LTE.

## Methods

All experiments employed the frequency-doubled (532 nm) output of a Nd:YAG laser, providing ≈70 mJ pulses with a Gaussian temporal profile and measured full width at half maximum (FWHM) duration of ≈7 ns. This laser light was focused at a 45° angle of incidence onto a rotating (1 rev min^−1^) SiC target (>99%, Testbourne Ltd., Basingstoke) mounted inside an ablation chamber maintained at ≈10^−7^ mbar. The spot size at the target surface was ≈5 × 10^−4^ cm^2^, implying an incident fluence *F* ≈ 150 J cm^−2^ and pulse-averaged irradiance *ϕ* ≈ 20 GW cm^−2^. The absolute pulse timings, energies, and durations were monitored continuously, as described previously^11^. Spatially resolved spectral images of the optical emission from the plasma were collected, using a time-gated intensified charge coupled device (ICCD) camera coupled to an imaging spectrograph (<0.1 mm spatial and 0.074 nm FWHM spectral resolution) and photomacrographic objective (overall magnification of unity; depth of field <5 mm), for different time delays Δ*t* after the ablation event. Time *t* = *t*_0_ was defined as the earliest at which plasma emission was observable, and Δ*t* and *t*_0_ were subject to <1 ns of shot-to-shot jitter. A 10 ns gate width, centered on (*t*_0_ + Δ*t*), was chosen to minimize expansion of the LIP during a measurement. Individual images were thus recorded over the interval *t* = *t*_0_ + Δ*t* − 5 ns to *t*_0_ + Δ*t* + 5 ns.

## Additional Information

**How to cite this article**: Liu, H. *et al*. Determination of Stark parameters by cross-calibration in a multi-element laser-induced plasma. *Sci. Rep*. **6**, 25609; doi: 10.1038/srep25609 (2016).

## Figures and Tables

**Table 1:** Stark parameters for the Si II, Si III, Si IV, and C II lines used in the present study, given for *N*_e_ = 10^23^ m^−3^.

Species	Transition	Wavelength / nm	Stark parameters	
			*w*/nm^ref^	*d*/pm^ref^
Si II	3s^2^4p – 3s^2^4s; ^2^P° – ^2^S	634.710*	**0.129**†, 15,25(0.133 ± 0.017)(0.123 ± 0.021)‡	**−45.1**^13^(**−**43.8 ± 4.8)(−43.2 ± 5.0)‡
		637.136	**0.134**†, 15,25	**−45.1**^13^
Si III	3s4p – 3s4s; ^3^P° – ^3^S	455.262*	**0.053** ± **0.004**^26^	**−10** ± **1**^26^
		456.784	**0.050** ± **0.005**^26^	**−8.0** ± **1.5**^26^
		457.476	**0.050** ± **0.005**^26^	**−9.0** ± **2.0**^26^
Si IV	2p^6^6h^1^ –2p^6^5g^1^; ^2^H° – ^2^G	465.432*	(0.194 ± 0.017)^11^	(**−**64 ± 8)^11^
C II	2s^2^3p – 2s^2^3s; ^2^P° – ^2^S	657.805*	**0.220**^27^ (0.203 ± 0.028)(0.206 ± 0.031)‡	**−11.0**^27^ (**−**14.0 ± 2.1)(−13.8 ± 2.9)‡

Entries in bold are literature values determined without assuming LTE, while values in parentheses have been derived by cross-calibration using the present method.

^*^These lines, also highlighted in Fig. 1(c,e), are those used for cross-calibration.

^†^Obtained by linear extrapolation of the literature values to *N*_e_ = 10^23^ m^−3^.

^‡^These values were obtained in a second, independent experiment.
