# Corrigendum: Determination of Stark parameters by cross-calibration in a multi-element laser-induced plasma

**DOI:** 10.1038/srep31015

**Published:** 2016-08-12

**Authors:** Hao Liu, Benjamin S. Truscott, Michael N. R. Ashfold

Scientific Reports 6: Article number: 25609; 10.1038/srep25609 published online: 05 12 2016; updated: 08 12 2016.

In this Article, the Data resources have been omitted from the Methods section. It should read:

“All data used in the preparation of this paper are available from the University of Bristol’s research data repository, and can be accessed using the DOI 10.5523/bris.rz5m2a3q61851txlxs6eecuhx”.

